# Mothers’ Sources of Child Fluoride Information and Misinformation From Social Connections

**DOI:** 10.1001/jamanetworkopen.2022.6414

**Published:** 2022-04-01

**Authors:** Jacqueline M. Burgette, Zelda T. Dahl, Janice S. Yi, Robert J. Weyant, Daniel W. McNeil, Betsy Foxman, Mary L. Marazita

**Affiliations:** 1 Departments of Dental Public Health and Pediatric Dentistry, School of Dental Medicine, University of Pittsburgh, Pittsburgh, Pennsylvania; 2 Center for Oral Health Research in Appalachia, Pittsburgh, Pennsylvania; 3 Department of Oral and Craniofacial Sciences, School of Dental Medicine, University of Pittsburgh, Pittsburgh, Pennsylvania; 4 Department of Dental Hygiene, School of Dental Medicine, University of Pittsburgh, Pittsburgh, Pennsylvania; 5 Department of Dental Public Health, School of Dental Medicine, University of Pittsburgh, Pittsburgh, Pennsylvania; 6 Department of Psychology, Eberly College of Arts & Sciences, West Virginia University, Morgantown; 7 Dental Public Health and Professional Practice, School of Dentistry, West Virginia University, Morgantown; 8 Center for Molecular and Clinical Epidemiology of Infectious Disease, School of Public Health, University of Michigan, Ann Arbor; 9 Department of Oral and Craniofacial Sciences, Center for Craniofacial and Dental Genetics, School of Dental Medicine, Pittsburgh, Pennsylvania; 10 Department of Human Genetics, Graduate School of Public Health, Pittsburgh, Pennsylvania; 11 Clinical and Translational Science, School of Medicine, University of Pittsburgh, Pittsburgh, Pennsylvania

## Abstract

**Importance:**

Fluoride delivered either topically (eg, fluoride varnish) or systemically (eg, water fluoridation, prescription fluoride supplements) is widely used as a caries preventive agent in children. The widespread existence of misinformation, including inaccurate information that attaches negative values or suspicion to fluoride treatments, raises concerns that misinformation will lead to underutilization of fluoride and contribute to avoidable increases in caries prevalence.

**Objective:**

To describe the social relationships from which mothers obtain child fluoride information and misinformation.

**Design, Setting, and Participants:**

This qualitative study analyzed 126 mothers with children aged 3 to 5 years to explore how their social networks were associated with their child’s oral health. Mothers were recruited from community-based sites in Pittsburgh, Pennsylvania, and throughout the state of West Virginia from 2018 to 2020. In-person, semi-structured qualitative interviews were conducted. Data were transcribed, coded, and analyzed using Nvivo 12. Three investigators analyzed data using template analysis, a qualitative technique that combines inductive and deductive approaches to identify patterns until primary themes are identified.

**Main Outcomes and Measures:**

Sources of information regarding fluoride from the mother's social relationships.

**Results:**

Of the 126 mothers with children aged 3 to 5 years in Pittsburgh and West Virginia, 120 (95%) identified as non-Hispanic White and 5 (4%) identified as Hispanic White; 38 (30%) had a bachelor’s degree, 77 (61%) had private dental insurance for their child, and 52 (41%) had an income less than $50 000; 82 (65%) reported that they discussed child fluoride information with members of their social network. Mothers described that they obtained fluoride information from family members, health care clinicians, and community members. The receipt of inconsistent child fluoride information from multiple sources resulted in confusion and difficulty assessing whether fluoride information was accurate.

**Conclusions and Relevance:**

This qualitative study found that mothers received inconsistent child fluoride information from multiple sources, which resulted in confusion and difficulty assessing the accuracy of the fluoride information. These findings suggest that social relationships can be a potential target for interventions to communicate fluoride information related to children.

## Key Points

**Question:**

From which social relationships do mothers obtain child fluoride information and misinformation?

**Findings:**

In this qualitative study of 126 mothers of children aged 3 to 5 years, fluoride information and misinformation relating to children were obtained from family members, health care clinicians, and community members. The receipt of inconsistent child fluoride information from multiple sources resulted in confusion and difficulty assessing the accuracy of the fluoride information.

**Meaning:**

These findings suggest that social relationships can be a potential target for interventions to provide accurate fluoride information related to children.

## Teaser

This qualitative study describes the social relationships from which mothers obtain child fluoride information and misinformation.

## Introduction

Nationwide, one of the most common preventable chronic childhood diseases is dental caries, commonly known as tooth decay.^1,2^ If left untreated, dental caries can lead to pain, infection, decreased school attendance and performance, and trouble eating, talking, socializing, and sleeping.^2,3,4,5^ Between 2015 and 2016 in the United States, approximately 21% of children aged 2 to 5 years experienced dental caries in their primary teeth.^6^

The Centers for Disease Control and Prevention describes the reduction of dental caries due to water fluoridation (the controlled addition of fluoride to community water supplies) to be one of greatest public health achievements of the 20th century: fluoride containing water is now delivered to more than 73% of the US population.^7,9,10^ Whether administered topically or systemically, fluoride can be an effective addition to comprehensive caries prevention or management intervention at individual and community levels.^2,7,8,9,10,11,12,13^ Guidelines support the application of fluoride varnish to the primary teeth of all infants and children starting at the age of primary tooth eruption.^14^ Fluoride’s mechanisms of action for caries prevention and management include its ability to decrease demineralization of enamel, increase remineralization of early carious lesions, and inhibit bacterial activity in dental plaque.^11^ There is extensive literature documenting that appropriate fluoride use is both safe and effective for preventing and managing dental caries.^7,8,9,10,11,12,13,14,15,16,17,18,19,20,21,22,23,24^

There are, however, both legitimate and illegitimate concerns regarding fluoride safety. The safety concerns range from minor cosmetic effects (eg, mild dental fluorosis) to concerns regarding serious medical issues (eg, cancer, neurotoxic effects) and are almost exclusively associated with ingestion of fluoride either intentionally (ie, via the introduction of fluoride into community water supplies, salt, or milk) or unintentionally through the inadvertent swallowing of topical fluoride products such as toothpaste and rinses. Fluorosis is linked to overingestion of fluoride during tooth formation,^21^ such as long-term ingestion of 1 to 10 ppm fluoride in the drinking water above the recommended 0.7 ppm fluoride concentration recommended by the US Public Health Service to prevent dental caries.^22^

More serious medical concerns have been suggested since widespread water fluoridation in the 1950s. However, for the most part, these concerns have been dismissed due to lack of supporting scientific evidence or the existence of strong evidence demonstrating that the concerns are, in fact, not valid. The most recent concern of note was raised in 2006,^25^ investigating if fluoride was associated with neurotoxic effects and further research was indicated. Several epidemiological studies followed that indicated an association.^26^ However, upon further in-depth reviews, these concerns were dismissed as being methodologically flawed and that neurotoxic effects would be unlikely to occur in typical community water fluoridation conditions found in high-income countries.^27,28,29,30^

Health decisions can become confusing when legitimate concerns are influenced by misinformation.^31,32,33,34^ Reports from pilot or poorly conducted studies suggesting adverse effects of fluoride have and continue to enter popular culture, raising fear and causing reluctance to use fluoride for caries prevention and management. Claims that fluoride causes cancer and limits cognitive development and thyroid functioning have been debunked,^35,36,37,38,39^ yet the fears may remain. Conflicting information can lead caregivers to choose to not expose their child to fluoride.^40^ There are multiple potential sources of fluoride misinformation. Online sources, such as social media, can persuasively present inaccurate information that quickly spreads, dilutes or discredits the advice of public health experts, and threatens public safety.^41^ Although there is published literature on how social media spreads fluoride misinformation,^42,43,44^ far less is known about the spread of misinformation through in-person social relationships. The purpose of this study was to fill this gap in how caregivers may be misinformed about fluoride by examining whether a portion of caregivers—mothers in Appalachia—obtained child fluoride information through their in-person social relationships.

## Methods

### Research Design, Data Collection, and Initial Analysis

In this qualitative study, we conducted one-on-one, in-person, semistructured interviews with mothers on how their social network affects child oral health. The participants were 126 mothers with children aged 3 to 5 years and recruited from a larger longitudinal parent study, the Center for Oral Health Research in Appalachia (COHRA2) from 2018 to 2020.^45^ This study was approved by the SMART Institutional Review Board mechanism with the University of Pittsburgh as the primary site and West Virginia University as the referring site. This study was conducted and reported in accordance with guidelines from the Standards for Reporting Qualitative Research (SRQR) reporting guideline.^46^ Written informed consent was obtained from all mothers. These interviews also are the basis for other research on child oral health.^47,48,49^

Fluoride was not included as a probe in our initial semistructured interview guide—which included domains on child dental visits, home dental hygiene and cariogenic diet—but arose as a common topic mentioned by the mothers. Three female interviewers collected qualitative data and tailored open-ended questions and prompts on child dental hygiene to information provided by the participants. These interviewers met as a team with the principal investigator bimonthly to review data, identify preliminary themes emerging from the interviews, and discuss additional domains to clarify in subsequent interviews.

### Researcher Characteristics and Reflexivity

The principal investigator (J.M.B.) is a clinician-scientist specializing in pediatric dentistry and health services research who experienced lack of access to fluoridated water as a child and provided fluoride information to families with young children. The research assistant (Z.T.D.) did not receive fluoride from a dentist until adulthood and often drank well water as a minor. The dental hygiene student (J.S.Y.) experienced poor access to dental care as a child, was exposed to fluoride misinformation as a layperson, received comprehensive education on fluoride during dental hygiene training, and serves disadvantaged communities that experience a burden of dental caries that may be reduced with fluoride.

### Data Analysis

The interviews were recorded, transcribed verbatim (TranscribeMe) and imported into NVivo version 12 (QSR International) to conduct template analysis. Template analysis uses deductive and inductive coding.^50,51^ We organized relevant data into a priori codes of “supportive of fluoride” or “unsupportive of fluoride” by relationship type. Additional codes emerged inductively during transcription analysis, such as “conflicting fluoride information from multiple sources.” A final codebook—including definitions, when and when not to use codes, and examples—was applied it to all qualitative data. We used the coded data to extract quotations for analysis. We wrote memos^52^ to summarize, analyze, and interpret why and how mothers sought information on child dental fluoride from their social relationships. Our final themes arose from the memos and were refined using a constant comparative method^53^ until theoretical saturation, or when no new ideas about the concepts emerge, was reached.^52^ An interviewer (Z.T.D.) for this study, provided field notes to aid in data interpretation. Data analysis was performed from November 2020 to June 2021.

## Results

In this qualitative study of 126 mothers, 120 (95%) identified as non-Hispanic White and 5 (4%) identified as Hispanic White; 38 (30%) had a bachelor’s degree, 77 (61%) had private dental insurance for their child, and 52 (41%) had an income less than $50 000 (Table 1). Two main themes emerged regarding how mothers obtain child fluoride information and misinformation from their social relationships. First, mothers obtained fluoride information from 3 types of social relationships: family members, health care clinicians, and community members. Eighty-two mothers (65%) received information on child dental fluoride primarily through these 3 types of social relationships (Table 1). Second, getting fluoride information from multiple sources was associated with confusion and difficulty assessing whether that information was accurate.

**Table 1.** Characteristics of Mothers in North and North-Central Appalachia Who Participated in the Semistructured Interviews on Their Social Networks Responsible for Child Oral Health^a^

Family sociodemographic characteristics	Mothers, No. (%) (N = 126)
Child age, mean (SD)	4.77 (1.02)
Child dental insurance	
Private	77 (61)
Public	18 (14)
None	31 (25)
Mother’s education	
≤High school or equivalent	18 (14)
Some college or associate degree	35 (28)
Bachelor’s degree	38 (30)
Master’s, doctorate or professional degree	35 (28)
Mother’s race and ethnicity	
Non-Hispanic White	120 (95)
Hispanic White	5 (4)
Other	1 (1)
Family income, $	
<50 000	52 (41)
50 000-99 999	47 (37)
≥100 000	20 (16)
Missing	7 (6)
Received information on child dental fluoride through the mother’s social network	82 (65)

^a^ The 126 mothers of children aged 3 to 5 years resided in Pittsburgh, Pennsylvania (n = 60) or West Virginia (n = 66).

### Familial Relatives

Mothers described that the most common source of child fluoride information was a family member (Table 2). One mother described that she inherited strong, long-standing fluoride support from her family, despite living far away from them. She stated, “My grandfather in [state] was an oral surgeon and led the fight to have our water fluoridated. So, my mom was always telling me that her father is rolling over in his grave about [me not having fluoridated water].” Her sister “made fun of [her] for living somewhere without fluoridated water” when she brought up needing fluoride supplements for her children and introducing fluoride toothpaste earlier than her sister’s children. After growing up in a family with complete support for fluoride treatment, the mother was surprised to find out her community’s water was not fluoridated, “because I didn’t know that even happened anymore until I lived there and had a child.”

**Table 2.** Select Quotations From Mothers on Child Dental Fluoride Information With Familial Relations

Family relation	Quote
Supporting fluoride use for children	
Grandfather	“But she said it’s just a kids’ fancy one with no fluoride. And I said, ‘Oh, no. You should get one with fluoride,’ because that’s the kind of lovely sister-in-law I am that I was like, ‘No, no, no. It’s really important.’ But that might have been influenced very much from my grandfather.”
Mother	“My mom did talk to me about fluoride treatments for the kids. They [my parents] believed in fluoride treatments.”
Father	“My mom didn’t want to do it because she said for millennia, kids have been drinking their parent’s breast milk without fluoride drops and have been just fine so breast milk has everything you need. My dad said, ‘Well, no, because I feel like I’ve been told they need fluoride drops. I’m going to do these drops.’”
Not supporting fluoride use for children	
Grandfather	“I feel like [distrust of fluoride] is always connected with people putting things in the water.”
	“I’ve never heard of people being against fluoride toothpaste. But I’ve always heard people say don’t put fluoride in my water. The closest somebody would have been would be one of my friends’ parents or something and they mentioned it in passing.”
	“I do have that one very opinionated grandfather that, everything the government does is wrong. It might have come from there and I just wrote it off because I’m like, ‘Oh, that’s just him.’”
Mother	“My mom talks about distrusting fluoride. We only got fluoride when [we] were kids. I don’t know if this is true or not true, but she said it put white spots on our teeth. I think she doesn’t so much maybe believe in giving the kids extra stuff that they don’t need. It’s kind of like ‘don’t give them medicine if they don’t need it’ kind of take on things.”
Extended family	“My family in Maryland. My cousin has an allergy to [fluoride] and they get special expensive filtered water without fluoride. And they also have a special head on their shower that does something with the fluoride in the water.”
	“And I’m even suspicious of it just because—I don’t know. Corporation. I don’t want to sound like a conspiracy theorist at all. I just don’t know the full history of why it’s added, why do we take it, and why it’s supplemented. And I guess, because I don’t have all the information. I’m not too willing to trust it just because somebody tells me I should. I know they do sell toothpaste without fluoride in it. I don’t know why or what the benefit is.”
Grandmother	“My daughter spends a lot of time with my great-grandmother. We call her ‘Baba,’ and she takes care of [my daughter] every day after school. She picks her up around noon, she goes home, they eat lunch. [My daughter] brushes her teeth every day at Baba’s before nap time. The only frustration I have there: I can’t get my Grandma on board with not using the training toothpaste. She kind of worries that [my daughter] doesn’t spit well enough. And that is just a worry of hers, even though we use the regular children’s toothpaste at home. But I think Grandma still uses the training toothpaste at her house, which, I mean, I guess is fine. Her teeth are still getting brushed and everything…I even bought a tube to send to her house. But I don’t think it’s getting used because I’ve seen it on the counter. But who knows? They might be transitioning. I’ve tried to ask, and she’s just like, ‘You just let me do me.’”

Mothers discussed that some family members supported dental fluoride for children and others opposed it (Table 2). One mother expressed her concern with fluoride after relaying her extended family member’s experience with a fluoride allergy. “My cousin has an allergy to [fluoride], and they get special expensive filtered water without fluoride. And they also have a special head on their shower that does something with the fluoride in the water.” The extent of her cousin’s sensitivity to fluoride requires the family to filter out fluoride from both their drinking and bathing water, raising fear and suspicion of fluoride safety, even though the mother is unaffected by a fluoride allergy. She sought additional fluoride information from her child’s dental hygienist to relieve her concerns and was unsatisfied when the hygienist referenced a trusted source without conveying her reasoning: “Well, because the FDA recommends it.” The mother described her lack of trust in the dental hygienist’s response by explaining that “I don’t have all the information. I’m not too willing to trust it just because somebody tells me I should” and “I just don’t know the full history of why it’s added, why do we take it, and why it’s supplemented.”

### Professional Relationships With Health Care Clinicians

As described in the aforementioned quote, mothers considered dental professionals—within the context of a professional health care relationship—as part of their social network for child oral health. Many mothers considered their health care clinicians as source of fluoride information for children (Table 3).

**Table 3.** Select Quotations From Mothers on Child Dental Fluoride Information From Health Care Clinicians

Health care clinician	Quote
Supporting fluoride use for children	
Dentist	“I also feel like they swallow such a huge amount of toothpaste when they brush their teeth that it’s okay they don’t [also have the fluoride tablets]. I don’t know if it’s available the same way as the tablets, but I did have a couple conversations with our dentist about it and she’s the one that told me about that eating or ingesting it is just as important as using it topically.”
Dentist	“I was thankful that her dentist, he was like, ‘Yeah. This age is tough, we have very low expectations. And cool, you got her in, she’s sitting on your lap in a chair. Win. That’s what we like. I’m happy to do a fluoride treatment if you want.’ I was like, ‘Yes. Let’s at least do that.’ And she flipped out, but it was quick enough that [it got done].”
Dentist	“As far as talking about my kids’ teeth, they’ll just say, ‘Oh they look good. The next time we may do this.’ I think [my son] has had a fluoride rinse, so I think they warned me, ‘In six months, if it’s okay with you we’ll probably do a fluoride rinse,’ and things like that.”
Pediatrician	“This was several years ago—but [the pediatrician] just said they’ve seen so many kids come in with just such awful teeth and bad dental-hygiene that it was such an importance to them to help these kids, and so they just made it uniform for everybody, just like a procedure now. So, that was how they presented it.”
Pediatrician	“Fluoride can cause cancer and all this stuff. So, I just recently asked my pediatrician about does he think that’s true. And then that brought up the whole conversation. He didn’t want to, ‘You just ask your dentist. We just—do what your dentist tells you if your dentist tells you to do it.’ But he said, ‘No. There’s nothing, right now, to say that it does cause cancers or anything or any problems.’ So, I settled down a bit. Okay.”
Pediatrician	“They don’t really recommend that you [use bottled water for babies], and it’s a touchy-subject because like, there’s lead in the water. So, what do you tell them? Drink the lead or have bottle of water with no fluoride. I will tell clients that I see using a bottle of water, ‘just so you know, you’re not getting that your child’s teeth aren’t getting fluoride when you’re doing that’. Our pediatrician told us a long time ago not to give them bottled water as the primary source of water that they’re getting.”
Dentist	“She will make recommendations that I don’t agree with or that I don’t want to hear, so. I mean, she recommends, like a normal dentist would, to do the fluoride stuff. I don’t put fluoride on my kid’s teeth. Amy’s regular recommendation is to do the fluoride treatment, and I don’t do it because it’s a toxic level of fluoride that they you put in your kid’s mouth. And I don’t know if that’s good.”
Not supporting fluoride use for children	
Dentist	“I actually drive past this one dentist that has a big sign on the front that says that they don’t use fluoride. It’s a fluoride-free dentist. And they do all sorts of natural things as well, but no fluoride. And people are really paranoid about the mercury thing, and so I think they have a sign about that, too. But we don’t go there, thankfully. We do not take our kids to that dentist. But it’s interesting that there is a dentist that their practice is pretty much for these people. They’re afraid of fluoride, mercury, all these things.”
Dentist	“Our water is not fluoridated, so the dentist did give him a prescription, and I did give him fluoride for a while. And then I switched dentists and I asked about that, and I feel like they said not to. And now, I’m thinking about, ‘Should my kids be getting fluoride or not?’ because they’re not. But I guess maybe it’s enough in the toothpaste now or not?”
Pediatrician	“Well, the pediatrician had said not to use fluoride toothpaste until they turned 3. I told the dentist because I had taken her early on, and it was fine as long as she knew to rinse and spit, and she did. She was really good about it.”
Midwife	“The only thing we didn’t do: I usually get the extra fluoride. It’s like a paste that they brush on. I didn’t do that while I was pregnant because I wasn’t sure if that was okay or not until I had talked to my midwife about it. But, you know, that was the only thing I didn’t do was the extra fluoride treatment during pregnancy. And, even my midwife wasn’t sure what the answer to that was.”
	“I was like, ‘Nobody knows. Now, I’d err on the side of caution and not do it;’ but her answer was basically, ‘Nobody likes to do tests on pregnant women. So, I don’t really have an answer for you.’”

One mother described that she received child fluoride information from both her child’s pediatrician and dentist: “Our pediatrician is the one that pointed out that … we didn’t have fluoride in our water. I learned that from my pediatrician … I feel like they swallow such a huge amount of toothpaste when they brush their teeth … I did have a couple of conversations with our dentist about it.” Once she knew her community water lacked fluoride, this mother spread this information throughout her social network, stating she “always tell other parents” and emphasized to one friend, “You have to [get fluoride supplements].”

Many mothers described being introduced to the benefits of fluoride for children’s teeth through the pediatrician’s application of dental fluoride varnish during a well visit. One mother reported that her pediatrician “would put fluoride on his teeth the first 2 years we were going. And then they said he needed to go to the dentist. So, we made the appointment.”

Mothers also cited dentists as a source of fluoride information, although they did not always agree with the dentists’ recommendations. One mother explained she did not believe fluoride is safe for children, but her dentist consistently offered fluoride treatments, despite her refusal. “She will make recommendations that I don’t agree with or that I don’t want to hear. So, I mean, she recommends, like a normal dentist would, to do the fluoride stuff. I don’t put fluoride on my kid’s teeth. [The dentist]’s regular recommendation is to do the fluoride treatment, and I don’t do it because it’s a toxic level of fluoride that they put in your kid’s mouth.”

In our results, mothers did not report any health care clinicians opposing child fluoride use. However, some pediatricians and dentists occasionally gave conflicting information about when to begin fluoride toothpaste and whether additional fluoride supplements were necessary (Table 3). Some mothers found their pediatrician reluctant to advise about child fluoride use, even when asking for reassurance about fluoride misinformation: “I heard something terrible about, ‘Oh, fluoride can cause cancer and all this stuff.’ So, I just recently asked my pediatrician about does he think that’s true. And then that brought up the whole conversation. He didn’t want to [address it], ‘You just ask your dentist. We just–do what your dentist tells you if your dentist tells you to do it.’ But then he said, ‘No. There’s nothing, right now, to say that it does cause cancers or anything or any problems.’”

In the aforementioned quotation, the pediatrician referred the mother to her dentist regarding questions about dental fluoride and cancer. Although the pediatrician stated that fluoride does not cause cancer, the mother also reported that the pediatrician did not want to talk about dental fluoride. Both referral and reluctance to discuss fluoride by a health care professional are important observations for a mother with serious concerns regarding carcinogenic effects.

### Community Members

Mothers described soliciting and receiving both profluoride and antifluoride information from community members (Table 4). Examples of profluoride information that mothers received included “common sense” child dental care tips from “people who had tried it” in a parent support group. Parents recommended that “if you’re concerned about cavities, make sure when they do start going to the dentist, they get fluoride treatments.” An example of antifluoride information came from a mother’s acquaintance who works for a water authority. He expressed strong disapproval of fluoride, stating, “It is disgusting. You shouldn’t have fluoride in your water. You should see the chemicals that I put in to fluoridate the water.” This mother dismissed the advice as “old-fashioned paranoia.”

**Table 4.** Select Quotations From Mothers on Child Dental Fluoride Information From Community Members

Community member	Quote
Supporting fluoride use for children	
Support group for mothers of children with attention deficit and hyperactivity disorder	“They’d say try different things, like try a softer toothbrush. Make sure it’s small. Dip it in some mouthwash if you’re uncomfortable with the fact—if you’re concerned about cavities, make sure that when they do start going to the dentist, they get fluoride treatments. Stuff like that. So, it was just kind of common-sense things that were coupled with people that had tried it.”
Friends	“Because when they first started doing [fluoride varnish at pediatrician visits], everybody [in my mom-group] was kind of like—‘What?’ People were trying to look it up, like ‘is this really what’s recommended?’ [and] we all found yeah, like they said, it is recommended.”
	“With all the [checkups at pediatrician], they always ask if we want to do the fluoride. We always say, ‘yeah’...I feel like generally among my mom-friends, that’s accepted. Everybody does that.”
Not supporting fluoride use for children	
Community	“I’ve never heard of people being against fluoride toothpaste, but I’ve always heard people say, ‘Don’t put fluoride in my water.’…It definitely wasn’t anybody close to me. The closest somebody would have been would be one of my friends’ parents or something and they mentioned it in passing.”
Community	“My husband and I researched a little bit about why isn’t our water fluoridated. And I think it was up for town debate on a township level at some point. And I can’t remember how long ago that was. But the decision was that it wasn’t the townships place to be medicating the public. They considered it a medication.”

In addition to recalling specific incidents of exchanging fluoride information from community members, mothers explained that they were aware of general beliefs about fluoride in the community, even if they did not know the detailed origin of those beliefs or how they may apply to children. For example, one mother stated, “I’ve never heard of people being against fluoride toothpaste, but I’ve always heard people say, ‘Don’t put fluoride in my water.’ … It definitely wasn’t anybody close to me. The closest somebody would have been would be one of my friends’ parents or something, and they mentioned it in passing.”

### Receiving Fluoride Information From Multiple Sources

Mothers may receive inconsistent fluoride information from multiple sources, complicating their decision to use fluoride on their child (Table 5). For example, one mother received profluoride information from a family member, health care clinician, and a community member, but chose not to adhere to any of the advice because she did not trust the sources. This mother drank fluoridated water as a child, and her exposure to excess fluoride led to “a lot of those white spots on my teeth growing up. And I had a lot, where I got made fun of. But I had my front 2 teeth shaved to get a lot of it removed.” Her dentist “did tell us to buy bottled water that has some fluoride in it because there’s not fluoride in our water. I’ve kind of ignored this advice so far.” Her pediatrician “told me that [their community water is not fluoridated] and I ignored it. And then I ignored it again.” In a conversation with a community member about fluoride, she maintained her antifluoride stance, “[My friend] would say, ‘Yes. Give them the fluoride’ … and I would still choose to ignore her and not do it for all the reasons that we just talked about.” This mother distrusted the fluoride information she received because of her traumatic childhood experience with fluorosis-affected teeth. The mother did not want her children to share her negative experience with fluorosis, so she withheld fluoridated water from her children. This example illustrates that the type of relationship and validity of the fluoride information may not be as important as the trust in the source.

**Table 5.** Fluoride Information From Multiple Sources

Social relationships	Quote
Supporting fluoride use for children	
Pediatrician	“Yeah. Well, the pediatrician had said not to use fluoride toothpaste until they turned three. And with my daughter, I started a lot earlier because I didn’t want a bad outcome again. So, I talked with their regular dentist and they told me about a certain child toothpaste—I had it ordered online. It just has extra fluoride or something in it.”
Friend	“We have this water filter, and our friend had it first, and they recommended it to us, and I didn’t buy the fluoride filters for it, then he really felt that I didn’t—that’s because fluoride is so bad for you, it can put holes in your brain, blah, blah, blah. But I like to give my kids fluoride water because well, that’s what they depend on until they’re going to the dentist and using fluoride toothpaste. So, my kids always had it…It’s not somebody I talk to all the time. And I was sitting there, and then I was kind of like, ‘I’m done with this.’”
Dental hygienist	“I know [a fluoride rinse] is good for them to use. My one girlfriend is a hygienist, and she told me that she uses it for [her children]…We both want to take care of our kids’ teeth. In fact, she is a hygienist, and she had the same exact issue with her daughter, and she’s had the same dental surgery done.”
Dentist and pediatrician	“[The dentist] did tell us to buy bottled water that has some fluoride in it because there’s no fluoride in our water. The pediatrician...probably told me that and I ignored it. And then I ignored it again.”
Sister and mother	“I’m sure my sister and my mom would recommend fluoride.”
Friend	“Well, if [my friend] was like, ‘Yeah, they should have fluoride, they should give them a lot of water.’ I mean, I don’t think that we ever have actually had this conversation. But I think that this is how it would go. She would say, ‘Yes. Give them the water with fluoride in it.’ And I would still choose to ignore her and not do it for all the reasons that we just talked about.”
Neighbor (nurse practitioner), pediatrician, and dentist	“I have asked the one neighbor about fluoride because we were trying to figure out if our water was fluoridated and it’s not, it turns out. And she is a nurse practitioner at [a university]. And she was saying that she gives her kids a fluoride supplement, which I had never heard of that. I mean, I never knew of that even when I was little. You just got the fluoride treatment at the dentist. So, I have that, actually, on my list to ask when we go to the pediatrician. I want to see if he thinks we should be on a fluoride supplement or whatever because I think she said they take it every day. But I had never heard of that because I’m like, ‘Well, don’t they just do the fluoride at the doctor now or at the dentist?’ And she said, ‘Well, they do have that. But we also give them a fluoride supplement.’”
Not supporting fluoride use for children	
Dentist	The mother asked dentists twice if they advise adding fluoride to her children’s routine, and the dentists did not provide profluoride information with the mother reporting that dentists “never said anything.”
Friend	Friend gifted the mother nonfluoridated toothpaste: “One of our friends bought us this [charcoal] toothpaste because it was black. So, she bought him that. It’s fluoride-free. We didn’t know [that] until after I had to go rebuy because it says fluoride-free [on the box].”

## Discussion

Mothers reported receiving child dental fluoride information from social connections, including family members, health care clinicians, and community members. At times, the messaging mothers received around fluoride safety and efficacy conflicted and caused difficulty and confusion when trying to reconcile it. This study’s results are consistent with previous literature documenting the spread of health-related misinformation in social networks,^54,55,56,57^ but add specificity to the types of relationships for child fluoride.

The results of this study have possible implications for clinical practice. Our findings suggest that clinicians can anticipate that caregivers may be receiving alternative messaging about fluoride from family and community sources. Therefore, clinicians may want to inquire whether caregivers have questions or concerns over the safety and efficacy of fluoride use. Listening to the mother’s concerns may help clinicians better understand any confusion around fluoride and then tailor their messaging in a way that addresses specific inaccurate information. Opening a discussion about the mother’s struggle to determine what is best for her child amid multiple conflicting pieces of fluoride information may engender more trust with clinicians. This family-centered approach to fluoride misinformation may help clinicians avoid a 1-way transfer of information, which may be ineffective in the face of multiple sources of fluoride information from trusted relationships.

This study has implications for the timing of child fluoride advice. Children may be unable to visit a dentist during the first few years of life,^58,59,60,61,62,63,64,65^ when home hygiene habits are established. In the absence of information on child fluoride from dentists, our results show that mothers may be receiving multiple sources of conflicting information about fluoride from other social relations, such as family and community members.

### Limitations

This study had some limitations. First, mothers were the exclusive focus in this study and were a convenience sample from a relatively racially and ethnically homogenous population in north and north-central Appalachia, so caution should be taken in generalization. Nevertheless, this population is one that bears the burden of oral health inequities, and so is a crucially important group of focus. Second, we did not ask mothers about fluoride information from social media, which previous studies have shown to include antifluoride information.

Future studies can examine effective communication and trust associated with child fluoride information. Researchers can examine the effectiveness of health communication theories and practices^66^ in the context of multiple fluoride messages from multiple sources. Future research can also include fathers and identify factors that engender trust in fluoride information. These factors could be related to how and why child fluoride treatment is beneficial and safe, the source, communication style, frequency, and timing of the message.

## Conclusions

This qualitative study found that mothers sought and received fluoride information from family, health care, and community members. These findings suggest that social relationships can both spread information and be a source of factual alternatives to misinformation on fluoride.
